# Case Report: CAR-T therapy for relapsed diffuse large B-cell lymphoma in a patient with pre-existing Parkinson’s disease—unfolding clinical challenges

**DOI:** 10.3389/fonc.2026.1759390

**Published:** 2026-02-26

**Authors:** Anas Ibraheem, Helena Vincentelli, Andrea Kuhnl, Emil A Kumar, Andres Moya Davila, Piers EM Patten, Deborah Yallop, Reuben Benjamin, Charlotte Graham, Aaron Niblock, Robin Sanderson

**Affiliations:** 1 Department of Haematology, King’s College Hospital, London, United Kingdom; 2 Department of Oncology, The London Clinic, London, United Kingdom; 3 Comprehensive Cancer Centre, School of Cancer and Pharmaceutical Medicine, King’s College London, London, United Kingdom; 4 Northern Health Care and Social Trust, Antrim, Northern Ireland, United Kingdom

**Keywords:** CAR-T therapy, diffuse large B-cell lymphoma (DLBCL), immune effector cell–associated neurotoxicity syndrome (ICANS), multidisciplinary approach, parkinsonism, Parkinson’s disease (PD)

## Abstract

Chimeric antigen receptor T-cell (CAR-T) therapy has transformed the outcomes for relapsed/refractory diffuse large B-cell lymphoma (DLBCL). However, immune effector cell–associated neurotoxicity syndrome (ICANS) remains a concern. Pre-existing neurological disorders such as Parkinson’s disease (PD) introduce additional, poorly studied challenges due to the risk of unpredictable complications. We report a 62-year-old man with relapsed DLBCL and pre-existing PD who was treated with lisocabtagene maraleucel. Baseline assessments by neurology, physiotherapy, and speech-language teams enabled tailored monitoring, including use of a modified Immune Effector Cell–Associated Encephalopathy (ICE) score. His dopaminergic regimen was optimised prior to therapy. Following lymphodepletion and CAR-T infusion, he experienced grade 1 cytokine release syndrome, which resolved with tocilizumab and ward-based supportive care. Although a stable partial response was observed on PET-CT scan at 1 and 3 months, the absence of ICANS or PD worsening up to his most recent follow-up on Day +86 suggests that CAR-T therapy can be safely delivered in patients with pre-existing PD when tailored strategies are applied, including a multidisciplinary approach, modified neurotoxicity monitoring, and careful selection of the CAR-T construct. Further studies and longer follow-up are needed to clarify long-term safety in this population.

## Introduction

Diffuse large B-cell lymphoma (DLBCL) is the most common aggressive non-Hodgkin lymphoma in adults. More than 60% of patients can be cured with R-CHOP (rituximab, cyclophosphamide, doxorubicin, vincristine, and prednisone) immunochemotherapy, even in advanced-stage disease. In contrast, patients with relapsed or refractory (R/R) disease often face unfavourable outcomes, particularly in early relapse (1–4). Chimeric antigen receptor T-cell (CAR-T) therapy, a gene-modified cellular treatment, has transformed the therapeutic approach for R/R DLBCL after at least two prior lines of treatment, and emerging data support its use in the second-line setting (5–7).

Despite these advancements, the toxicity profile of CAR-T cells poses significant clinical challenges, most notably cytokine release syndrome (CRS) and immune effector cell–associated neurotoxicity syndrome (ICANS) (8). The latter represents a wide range of neurological manifestations, ranging from mild cognitive deficits, paligraphia, and paragraphia to seizures, encephalopathy, aphasia, and cerebral oedema (9, 10). Thus, pre-existing neurological disorders such as Parkinson’s disease (PD) introduce additional, poorly studied challenges due to the risk of unpredictable complications and their exclusion from pivotal clinical trials. PD symptoms can fluctuate under stressors such as infection, inflammation, and dopamine-blocking medications commonly used for antiemesis or delirium may precipitate severe deterioration (11). Moreover, the reported cases of Parkinsonism following CAR-T therapy highlight concern that patients with pre-existing PD may be at risk of relapse or worsening of their symptoms (12–14).

To our knowledge, this is the first reported case of CAR-T therapy in a patient with relapsed DLBCL and pre-existing PD. It underscores the practical challenges of delivering CAR-T in this population and the strategies used to mitigate neurological risk while addressing the underlying lymphoma.

## Case presentation

A 62-year-old right-handed man with PD since 2016 was referred to our tertiary centre for CAR-T therapy after relapsed DLBCL. He initially presented in August 2024 to his local hospital with right-leg swelling due to an ipsilateral inguinal mass and deep vein thrombosis. Subsequently, the mass biopsy confirmed the diagnosis of DLBCL.

Between September and December 2024, he received six cycles of polatuzumab, rituximab, cyclophosphamide, doxorubicin, and prednisone (Pola-R-CHP). His end-of-treatment imaging in February 2025 showed a partial metabolic response (Deauville 4), with a residual 9cm right pelvic mass. Clinically, he was independent in activities of daily living, able to drive short distances and prepare meals, with an ECOG performance status of 1 and Karnofsky score of 70.

In April 2025, a disease relapse was confirmed by biopsy of a recurrent right inguinal mass. At this point, he was referred to our tertiary centre for consideration of CAR-T therapy. He underwent apheresis in June 2025 and received bridging radiotherapy to the pelvic and inguinal nodes. Pre-lymphodepletion PET-CT scan in July 2025 revealed FDG-avid lymphadenopathy in the inguinal, iliac, retroperitoneal, and peritoneal regions (Deauville 4). Baseline brain MRI was limited by motion artefact; however, no gross intracranial abnormality was identified on interpretable sequences.

Given the known risk of CAR-T-related neurotoxicity and the patient’s pre-existing Parkinson’s disease, several measures were implemented as part of pre-CAR-T risk stratification. His baseline was assessed by a neurologist, a physiotherapist, and a speech-language therapist (SALT), who provided tailored monitoring and actively followed him throughout the admission. Neurological examination revealed mild cognitive impairment, bradykinesia, mild rigidity, truncal dystonia, resting tremor predominantly affecting the right side, and hypophonic dysarthrophonia with a longstanding stutter. He used a cane, although it was not necessary for walking short distances. His admission Parkinson’s regimen consisted of carbidopa/levodopa (Sinemet^®^) 25/100 mg 5 times a day, as well as co-careldopa controlled release (CR) 50/200 mg nightly, and safinamide 50 mg twice daily as adjunctive therapy for motor fluctuations. To optimise dopaminergic support during admission, opicapone 50 mg nightly was initiated. A contingency plan was agreed upon for alternative dopaminergic delivery (dispersible formulations or a rotigotine patch) if he becomes nil by mouth or is unsafe to tolerate a solid diet.

The physiotherapy assessment established a clear baseline and emphasised strategies to prevent deconditioning during admission, including twice-daily exercise, safe mobilisation with supervision if orthostatic hypotension occurred, and routine out-of-bed activity. Importantly, he was educated on the interaction between PD and post-infusion hypotension and on how to manage dizziness safely on the ward. SALT review noted a long-standing stammer and mild hypokinetic dysarthrophonia, both of which reduced speech intelligibility, especially with unfamiliar listeners. The team created communication guidelines for the clinical team and developed strategies to assist patients with communication, including laminated cue cards as needed. These measures ensured reliable neurotoxicity monitoring despite his baseline deficits.

After completing three days of lymphodepleting chemotherapy with fludarabine (30 mg/m²) and cyclophosphamide (300 mg/m²), the patient had a two-day rest before receiving lisocabtagene maraleucel (Liso-cel; Breyanzi^®^) on July 29, 2025. An in-specification product was infused at a concentration of 1.1–7.0 × 10^6 CAR+ viable T cells/mL, comprising a target 1:1 ratio of CD4+ and CD8+ components. Immediately post-infusion, he developed transient asymptomatic hypotension, managed with intravenous fluids.

From Day +1 to +6, the patient remained clinically well, with no evidence of CRS or ICANS, and consistently maintained an Immune Effector Cell–Associated Encephalopathy (ICE) score of 10/10. ICE assessments were performed twice daily starting on Day 0 and more frequently as clinically indicated. On Day +7, the patient developed an isolated fever without hypotension, consistent with grade 1 CRS as per the American Society for Transplantation and Cellular Therapy (ASTCT) grading criteria (15). Broad-spectrum antibiotics were commenced, and the fever resolved with paracetamol. Later the same day, he experienced transient hypotension in the absence of fever or hypoxia. As these features did not occur concurrently, CRS remained grade 1. A single dose of 8 mg/kg intravenous tocilizumab was administered due to evolving clinical concern, with rapid clinical improvement; neurological status remained stable. Cultures remained negative; therefore, all antibiotics were stopped over the subsequent couple of days. Notably, he required intermittent intravenous fluid boluses for hypotension but no vasopressor support. The patient was discharged well from the hospital on Day +28. No ICANS occurred throughout his admission, and ICE assessments were adapted with the use of shape-drawing tasks due to his baseline dysarthria and tremor.

**Figure 1:**
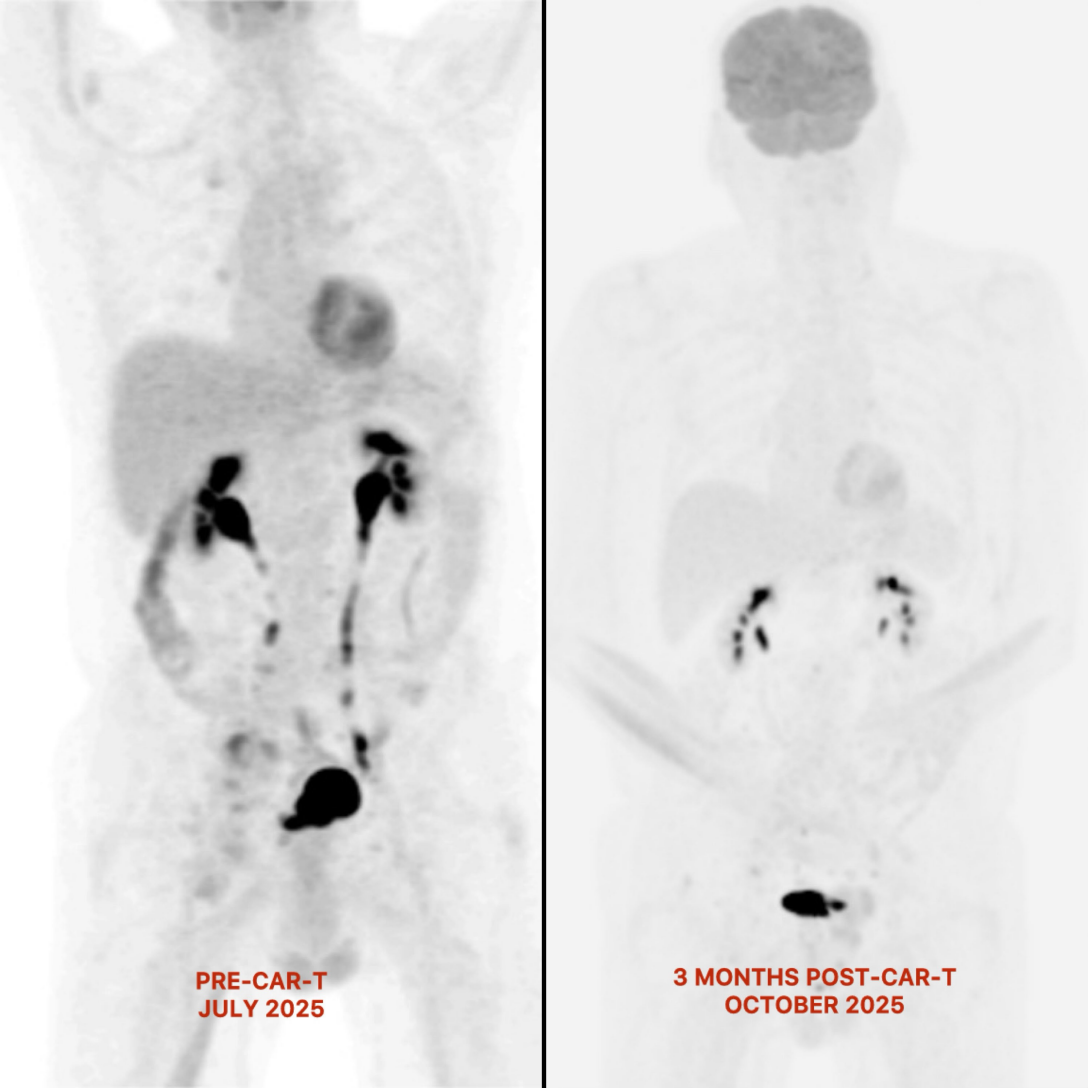
Changes in metabolic activity pre- and post-CAR-T therapy on PET-CT scan. No residual hypermetabolic nodular disease in the retroperitoneum or mesentery, with a marked bulk reduction in the right inguinal and common iliac lymphadenopathy 3 months after CAR-T therapy.

He has tolerated liso-cel remarkably well, without neurotoxicity or worsening of his underlying PD. Since returning home, his performance status has returned to baseline, and he remains active, walking his dog daily. Although the 1-month post-CAR-T PET-CT scan showed a partial metabolic response with borderline avidity, the patient was clinically well and expressed a preference to allow more time for the CAR-T effect to take hold. Upon reflection, this was appropriate, as the repeat PET-CT scan at 3 months post-infusion showed very stable findings, with borderline avidity within the radiotherapy field and a reduction in the size of the abdominal lymph nodes (**Figure 1**). It is more reassuring to see a stable Deauville 4 response at 3 months, consistent with a CAR-T response. Therefore, the CAR-T multidisciplinary meeting recommended continued observation, with a repeat PET-CT scan planned at 6 months post-infusion rather than considering further therapies.

## Discussion

In comparison to the conventional salvage chemoimmunotherapy followed by autologous stem-cell transplant as a second-line therapy for R/R DLBCL, the TRANSFORM and ZUMA-7 trials demonstrated that liso-cel and axicabtagene ciloleucel (Axi-cel) significantly improved event-free survival and response rates on extended follow-up (5, 6). These findings established CAR-T as a transformative option for patients previously considered to have a poor prognosis. Despite these advances, toxicity remains a significant challenge. ASTCT grading criteria and multidisciplinary management algorithms have improved the recognition and treatment of CRS and ICANS; yet, the incidence remains substantial and unpredictable across products and diseases (15, 16). Meta-analyses estimate that all-grade ICANS occur in 26.9% of recipients with haematological malignancy (with ~10% being high-grade), mandating vigilant neurological assessment in at-risk populations (17).

The interface between CAR-T neurotoxicity and pre-existing neurological disease is a surfacing area of concern that remains poorly defined, with limited prospective data. Existing literature suggests that baseline neurological comorbidity should not automatically preclude the use of CAR-T. However, it may complicate presentation, monitoring, and recovery from ICANS. Additionally, evidence from risk-modeling studies has yielded mixed conclusions regarding whether antecedent cognitive/neurological impairment independently elevates ICANS risk. This uncertainty underscores the need for individualized risk stratification and proactive mitigation (18–22). Additionally, the incidence of Parkinsonism post-CAR-T therapy highlights concerns that patients with pre-existing PD may be at risk of relapse or worsening of their symptoms (12–14).

While Parkinsonism after CAR-T has been linked to BCMA-targeted constructs via ectopic antigen expression in the nervous system, this rationale is not relevant to CD19-directed products such as liso-cel (12). More broadly, inflammatory cytokines surge (IL-6, IL-1, IFN-γ, etc.) and endothelial activation can disrupt the blood–brain barrier (BBB), amplifying microglial/astrocytic activation and impairing dopaminergic circuitry, leading to ICANS and Parkinsonism presentations (8, 9, 19). In this patient, liso-cel was deliberately chosen because of its 4-1BB costimulatory domain and a defined CD4+:CD8+ ratio, which are associated with slower expansion, lower cytokine peaks, and reduced neurotoxicity compared with CD28-based constructs such as axi-cel, owing to less endothelial activation, reduced BBB permeability, and less microglial overactivation (23, 24).

This patient achieved a stable partial metabolic response post-CAR-T without early relapse, and the absence of ICANS does not necessarily indicate inadequate CAR-T expansion. Multiple studies have demonstrated that CRS, ICANS, or tocilizumab administration does not adversely affect CAR-T efficacy, cell expansion, or persistence (25–27). Moreover, liso-cel has consistently been associated with a lower risk of ICANS than CD28-based constructs (23, 24), supporting the interpretation that the absence of ICANS in this case was more likely due to deliberate product selection rather than insufficient CAR-T expansion. While limited expansion cannot be entirely excluded in the absence of a direct biomarker, the absence of ICANS or PD worsening in our patient is more plausibly explained by a combination of construct choice and tailored supportive strategies, rather than lack of CAR-T activity alone, particularly given the patient’s stable response post-infusion. Nonetheless, further studies are needed to clarify these mechanisms.

Additional strategies for safe delivery of CAR-T therapy included a proactive, multidisciplinary approach to establish a neurophysical baseline and a supportive plan to maintain motor and cognitive stability (18). A modified ICE score tailored to his neurological deficits was adopted. Sequential shape-drawing was used in place of sentence writing (e.g., square → circle → square → triangle) to reduce linguistic and motor confounders inherent to PD, while preserving sensitivity to visuospatial and executive dysfunction relevant to ICANS. Meanwhile, the remaining tasks of the classical ICE score were maintained without alteration (i.e., assessment of orientation, attention, and command-following). This tailored approach, consistent with recommendations by Kazzi et al., is used when factors affecting the reliability of the conventional ICE score are present, including inadequate motor, auditory, and visual sensory function, as well as a language barrier (28).

Collectively, these strategies enabled the safe delivery of CAR-T therapy without ICANS or PD exacerbation. Our observation period extended to Day +86, during which PD remained stable. However, as cases of CAR-T–associated Parkinsonism have been described at later time points (approximately Day +19 to Day +100 post-cell infusion) (12, 29), our findings cannot exclude delayed neurological complications, and longer follow-up is necessary to establish long-term safety in this population. Formal neurological scoring tools (Unified Parkinson’s Disease Rating Scale part III [UPDRS-III], Mini-Mental State Examination [MMSE], or Montreal Cognitive Assessment [MoCA]) were not performed in this case; neurological stability was assessed clinically through serial examinations and daily functional status. Future cases would benefit from standardised assessments to strengthen objective longitudinal evaluation.

The central nervous system is a highly integrated, immunologically active network, in which dysregulated peripheral immune responses can exacerbate neuroinflammation. In PD and other neurodegenerative disorders, an imbalance between pro-inflammatory effector T cells and neuroprotective regulatory T cells (Tregs) has been implicated in disease progression; non-selective lymphodepletion and early CAR-T expansion may, in theory, exacerbate this imbalance. Emerging preclinical data suggest that Treg-enhancing strategies, including cytokine modulation or adoptive cellular approaches, may attenuate neuroinflammation and support neurological stability (30, 31). Although investigational and not applied here, such methods may mitigate ICANS, but they also carry the risk of impairing CAR-T efficacy. Future studies are needed to optimise this balance in patients with pre-existing neurodegenerative disease.

## Conclusion

This case demonstrates that CAR-T therapy can be safely delivered in patients with pre-existing PD when tailored strategies are applied to reduce the risk of neurological deterioration and ICANS. Pre-CAR-T risk stratification is a critical first step. Key measures included the deliberate selection of a 4-1BB–based CAR-T construct, a multidisciplinary approach, modified ICE scoring for accurate neurotoxicity monitoring, and optimised dopaminergic support. This experience highlights that patients with PD should not be categorically precluded from CAR-T therapy. Nevertheless, further case reports with longer follow-up are required to assess delayed effects, and larger studies are needed to validate these findings in broader populations.

## Data Availability

The original contributions presented in the study are included in the article/supplementary material. Further inquiries can be directed to the corresponding author.
